# Angiotensin-(1-7) relieved renal injury induced by chronic intermittent hypoxia in rats by reducing inflammation, oxidative stress and fibrosis

**DOI:** 10.1590/1414-431X20165594

**Published:** 2017-01-09

**Authors:** W. Lu, J. Kang, K. Hu, S. Tang, X. Zhou, S. Yu, L. Xu

**Affiliations:** Division of Respiratory Disease, Renmin Hospital of Wuhan University, Wuhan, China

**Keywords:** Ang(1-7), Renal injury, Obstructive sleep apnea, Inflammation, Oxidative stress, Fibrosis

## Abstract

We aimed to study the renal injury and hypertension induced by chronic intermittent hypoxia (CIH) and the protective effects mediated by angiotensin 1-7 [Ang(1-7)]. We randomly assigned 32 male Sprague-Dawley rats (body weight 180-200 g) to normoxia control, CIH, Ang(1-7)-treated normoxia, and Ang(1-7)-treated CIH groups. Systolic blood pressure (SBP) was monitored at the start and end of each week. Renal sympathetic nerve activity (RSNA) was recorded. CTGF and TGF-β were detected by immunohistochemistry and western blotting. Tissue parameters of oxidative stress were also determined. In addition, renal levels of interleukin-6, tumor necrosis factor-α, nitrotyrosine, and hypoxia-inducible factor-1α were determined by immunohistochemistry, immunoblotting, and ELISA. TUNEL assay results and cleaved caspase 3 and 12 were also determined. Ang(1-7) induced a reduction in SBP together with a restoration of RSNA in the rat model of CIH. Ang(1-7) treatment also suppressed the production of reactive oxygen species, reduced renal tissue inflammation, ameliorated mesangial expansion, and decreased renal fibrosis. Thus, Ang(1-7) treatment exerted renoprotective effects on CIH-induced renal injury and was associated with a reduction of oxidative stress, inflammation and fibrosis. Ang(1-7) might therefore represent a promising therapy for obstructive sleep apnea-related hypertension and renal injury.

## Introduction

Obstructive sleep apnea (OSA) is a chronic disease that influences several systems *in vivo*. It is characterized by the obstruction of the upper airways with repetitive pauses in breathing during sleep (despite efforts to breathe) as well as by daytime sleepiness (1). OSA affects at least 2–4% of the adult population, largely impacting older adults, and its prevalence has been reported to exceed 30% in those aged 65 years and above (2). In addition, approximately 50% of patients are hypertensive and an estimated 30-40% of hypertensive individuals have OSA (3), with OSA representing by far the most common secondary condition associated with resistant hypertension (4). Notably, Fletcher (5) has emphasized the causal link between chronic intermittent hypoxia (CIH) and increased arterial pressure. The relationship between OSA and hypertension has been extensively investigated by other research as well (4,6). Patients with severe OSA also exhibit a high prevalence of chronic kidney disease (CKD) and it has further been found to accelerate the loss of kidney function. Moreover, animals exposed to intermittent hypoxia suffer histopathological renal damage (7). However, the underlying mechanism of this association remains unclear despite the progress made by many studies in understanding OSA and renal injury.

OSA might contribute to renal disease through its association with vascular dysfunction and inflammation, oxidative stress, intra-renal hypoxia, dysregulation of the renin-angiotensin-aldosterone system (RAAS), and increased sympathetic nervous system activity (8–10). It has been found that activation of angiotensin II (Ang II) plays an important role in the pathological and physiological process of CIH (11). RAAS activation is implicated in most forms of kidney injury and inhibiting its main effector, Ang II, remains a cornerstone of therapy (12,13). Angiotensin-converting enzyme-2 (ACE2) efficiently hydrolyzes Ang II into angiotensin 1-7 [Ang (1–7)], a bioactive peptide in the renin-angiotensin system that has regulatory actions counter to those of AngII (14). It was proposed that the activation of the ACE2-Ang(1–7)-Mas axis might prevent or reverse organ damage in experimental models of renal diseases (15). Consistent with this, Ferrario et al. (16) demonstrated that increased Ang(1-7) could serve as a renoprotective factor. However, the role of Ang(1-7) in the CIH-exposed kidney remains unknown. In the present study, our aim was to explore the mechanism of OSA and renal injury and the potential protective role of Ang(1-7) in CIH-induced hypertensive rats.

Specifically, the present study was designed to test the hypothesis that a 4-week treatment with Ang(1-7) could exert renoprotective and antihypertensive effects in CIH rats, an OSA animal model that displays a progressive development of sleep apnea (17). We focused on the effects of Ang(1-7) treatment at various levels as indicated by oxidative stress markers, structural modifications (renal fibrosis), inflammation markers, and renal apoptosis.

## Material and Methods

### Experimental protocols

Male Sprague-Dawley rats (n=32, body weight=180-200 g) were purchased from the Experimental Animal Center of Wuhan University (Wuhan, China). Eight rats (exposed to normoxia control, Norm group) were randomly selected to establish the baseline levels of all the biomarkers. The remaining 24 rats were subcutaneously embedded with an osmotic mini-pump (Alzet model 2004, Cupertino, USA), and randomly divided into three groups, denoted as CIH (CIH exposure without any treatment), CIHAng (CIH exposure+Ang(1-7) infusion at 400 ng·kg^-1·^min^-1^ for 28 days) or NormAng (normoxia exposure+Ang(1-7) infusion at 400 ng·kg^-1^·min^-1^ for 28 days).

This study was approved by the Ethics Committee of Wuhan University, China, and conducted in accordance with the Declaration of Helsinki and the Guide for the Care and Use of Laboratory Animals, as adopted and promulgated by the United National Institutes of Health. The rats were housed in departmental animal chambers and were maintained under a 12-12 h light-dark cycle under standard laboratory conditions (temperature, 25±2°C; humidity, 60±5%). The rats were provided standard rodent chow and allowed free access to water. At the end of the experiment, all rats were sacrificed as described below. Every effort was made to minimize the number of rats used and their suffering during the experiments.

The model of CIH was established according to previously published methods (18). Briefly, sealed chambers were used to generate a hypoxic environment. Pure nitrogen and compressed air were distributed into each chamber through timed solenoid valves. Using 90-s cycles, pure nitrogen was infused into each chamber for the first 30 s until the minimum oxygen concentration reached 5%. Compressed air was infused for the remaining 60 s to allow the oxygen concentration in the chambers to return gradually to 20.9%. For the Norm group, air was forced into the chamber. For CIHAng and CIH groups, hypoxia exposure experiments were performed between 8:00 am and 4:00 pm.

### Measurement of SBP

Systolic blood pressure (SBP) was measured every week using tail cuff plethysmography (RBP-1 non-invasive BP analyzer, Chengdu Technology, China), in conscious animals. The average of three measurements was used for data analysis.

### RSNA recording

Renal sympathetic nerve activity (RSNA) was recorded on rats under deep isoflurane anesthesia using fine wire bipolar electrodes placed on the left renal nerve under direct visualization using a surgical microscope. A left subcostal incision was made and the kidney was approached from the retroperitoneal space under an anatomical microscope. A bundle of renal nerves was identified and gently freed from the surrounding tissue. One of the renal nerves was dissected and hooked to a pair of silver electrodes. To insulate the electrodes and the nerve from the surrounding tissue and to prevent desiccation of the nerves, we covered the electrodes and the nerve preparation with liquid paraffin. Electrical changes in RSNA were amplified, filtered, and monitored on an oscilloscope with a low-frequency cutoff of 100 Hz and a high-frequency cutoff of 3000 Hz. RSNA was integrated at a time constant of 10 ms with a sampling frequency at 10 kHz. Rats were allowed to stabilize for 30–60 min after surgery before initiating the acute experimental protocols. The bipolar platinum electrode was connected to a biological polygraph (RM6240BD, Chengdu Technology, China) to record RSNA simultaneously. After stabilization of the signal, the rats of the CIHAng and NormAng groups were given a daily dose of Ang(1-7) (576 µg/kg) via intravenous injection into the tail vein, and changes in RSNA were observed. Integrated RSNA was simultaneously recorded. The postmortem background signal was determined and the experimental data were corrected for this value. The change in RSNA after the intervention is reported as the percent change from the baseline.

### Collection of tissue samples and histological study

Rats from each experimental group were sacrificed via blood collection through intracardiac puncture. Blood samples were subjected to centrifugation at 377.3 g for 15 min at 4°C and the sera were stored at −80°C. Both kidneys of each animal were perfused with saline solution through the abdominal aorta until they were free of blood. For histological and immunohistochemical studies, decapsulated kidneys were cut longitudinally, fixed in phosphate-buffered 10% formaldehyde, pH 7.2, and embedded in paraffin. Then, 3-µm sections were cut, stained with Sirius red, and illuminated with a polarized light. Histological observations under light microscopy were performed using a Nikon E400 light microscope (Nikon Instrument Group, USA). Measurements were carried out with Image-Pro Plus image analyzer version 4.5 for Windows (Media Cybernetics, Silver Spring, MD). The remaining parts of the renal tissues were immediately frozen in liquid nitrogen and stored at −80°C.

### Immunohistochemical analysis

Kidney samples were fixed in 10% neutral formalin and embedded in paraffin, and then 3-mm thick sections were cut. The sections were incubated twice in xylene for 5 min each, followed by dehydration in a gradient of histology-grade ethanol for 5 min each, then rehydrated and rinsed in phosphate-buffered saline (PBS). Slides were then incubated in 30% H_2_O_2_ for 10 min at room temperature and washed twice with distilled water and PBS. Bovine serum albumin was added to the slides for 15 min. Next, the excess serum was removed and one of the following primary antibodies was added (all antibodies were from Boster, China, unless otherwise indicated): goat polyclonal anti-IL-6 (1:200 dilution), monoclonal antibody against rat TNF-α (dilution 1:50), monoclonal antibody against anti-HIF-1α (dilution 1:100), polyclonal antibody anti-CTGF (1:100 dilution), rabbit polyclonal antibody anti-nitrotyrosine (dilution 1:100; Millipore, USA) or polyclonal antibody anti-TGF-α (1:100 dilution), followed by incubation for 1 h at room temperature. After three washes with PBS for 3 min each, secondary antibodies (goat anti-rabbit IgG-HRP, 1:50,000, Boster) were added and incubated for 15 min at room temperature, followed by three 3-min washes with PBS. Integrated optical density (IOD) was measured using Image Pro Plus (version 6.0; Media Cybernetics, USA).

### Measurement of plasma levels of TNF-α, IL-6, and Ang II

Blood samples were centrifuged at 1756 *g* for 10 min and stored at -20°C until use. Plasma levels of TNF-α, IL-6, and Ang II were detected by solid-phase sandwich enzyme-linked immunosorbent assay (ELISA) kits (Elabscience Biotechnology Co., Ltd., China) specific for these factors and absorbance was measured at 450 nm using a plate reader (BioTek ELx800, USA).

### Measurement of oxidative parameters in the renal tissue

Frozen renal specimens from the rats were homogenized in tissue lysis buffer (Beyotime, China). After lysis for 15 min on ice, the homogenates were centrifuged at 1756 *g* for 15 min at 4°C. The malondialdehyde (MDA) contents in the supernatant were measured using commercially available kits (Jiancheng Bioengineering Institute, China). Briefly, the MDA contents in the homogenates were determined spectrophotometrically by measuring the presence of thiobarbituric acid-reactive substances as follows: 3 mL 1% phosphoric acid and 1 mL 0.6% thiobarbituric acid solution were added to 0.5 mL plasma that had been pipetted into a tube. The mixture was then heated in boiling water for 45 min. After the mixture had cooled, the color was extracted into 4 mL n-butanol. Absorbance was measured using a spectrophotometer (UV-1601; Shimadzu, Japan) at 532 nm. The amount of lipid peroxide was calculated to represent the thiobarbituric acid-reactive substances subject to lipid peroxidation. The results are reported in nmol/mg protein, according to a standard graph prepared from measurements of standard solutions (1,1,3,3-tetramethoxypropane).

The activity of SOD in the renal tissues was measured using a commercial assay kit (Jiancheng Bioengineering Institute), following the manufacturer's instructions. Briefly, this assay kit employs a thiazole salt for the detection of superoxide anions by producing a colored product. The absorbance was measured at a wavelength of 450 nm. One unit of SOD was defined as the total enzyme needed to produce 50% dismutation of superoxide radicals. To assay catalase (CAT), 100 µL kidney homogenate was diluted to a total volume of 1.2 mL with sodium phosphate buffer (50 mM), pH 7.0, and mixed with 1 mL H_2_O_2_ solution (30 mM). The IOD of each sample was measured at 240 nm for 3 min against a reagent blank containing buffer instead of kidney homogenate. CAT values were reported as the absorbance at 405 nm. An enzyme activity unit was defined as the degradation of 1 μmol H_2_O_2_·s^-1^·mg^-1^ protein. The enzyme activity is reported as units/mg protein.

### Western blotting

Protein concentrations were measured using the bicinchoninic acid protein assay (Thermo Scientific, USA). Equal amounts of boiled protein (40 μg) in the loading buffer were separated via NuPAGE 10% Bis-Tris sodium dodecyl sulfate-polyacrylamide gel electrophoresis (Life Technologies, USA) and then electrophoretically transferred to polyvinylidene fluoride membranes (Millipore). The membranes were subsequently incubated with primary antibodies (anti-TGF- α antibody, 1:800 dilution, bioWORLD, USA; anti-CTGF antibody, 1:1,000 dilution, Cell Signaling Technology (CST), USA; anti-HIF-1α, 1:1,000 dilution, Abcam, UK; cleaved caspase 3 antibody, 1:1,000, dilution, CST; and cleaved caspase 12 antibody, 1:1,000 dilution, Proteintech Group, Inc., USA), washed three times with TBST buffer (10 mM Tris-HCl, 0.15 M NaCl, and 0.05% Tween 20, pH 7.2), and incubated at room temperature for 1 h in the presence of horseradish peroxidase-conjugated secondary antibody (goat anti-rabbit IgG, 1:50,000; Boster). After the blots were washed three times with TBST buffer, they were developed and exposed using an enhanced chemiluminescence system on Hyperfilm X-ray films. The resultant protein bands were quantified by densitometry (QuantityOne 4.5.0 software; Bio-Rad Laboratories, USA).

### Terminal deoxynucleotidyl-transferase-mediated dUTP digoxigenin nick end labeling (TUNEL) assay

Apoptosis was evaluated via the *in situ* TUNEL assay, according to the manufacturer’s instructions (Roche, USA). Serial sagittal sections of the kidney were digested with 20 μg/mL proteinase K (Dako, Denmark) for 15 min and then immersed in 3% hydrogen peroxide for 5 min and incubated with terminal deoxynucleotidyl transferase at 37°C for 1 h. The sections were subsequently incubated with an anti-digoxigenin-peroxidase antibody at 37°C for 30 min,visualized with diaminobenzidine, and counterstained with hematoxylin. Detection of apoptotic cells was performed manually under a light microscope at a magnification of 200×. The apoptotic index was calculated as the percentage of cells showing TUNEL positivity. TUNEL (+) cells were counted from five random fields for each rat.

### Statistical analysis

Data are reported as means±SE. Statistical analysis was performed using SPSS V17.0 software (SPSS, USA). Statistical comparisons between groups were conducted with one-way analysis of variance (ANOVA) and the least significant difference test was performed for multiple comparisons. Change of RSNA was determined by an independent sample *t*-test. A value of P*<*0.05 indicated a significant difference.

## Results

### SBP

**Figure 1:**
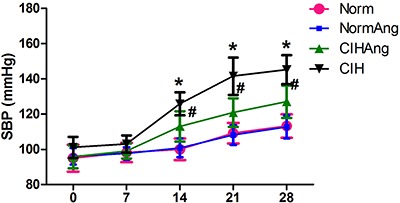
Systolic blood pressure (SBP) in Sprague-Dawley rats in the four groups (n=8). Norm: normoxia control group; NormAng: Norm and Ang(1-7) supplemented group; CIH: chronic intermittent hypoxia; CHIAng: CIH exposure+Ang(1-7) infusion. Data are reported as means±SE. During the experimental days: *P=0.0001, CIH *vs* CIHAng rats; 21 and 28 days: *P<0.0001, CIH *vs* CIHAng rats; 14 days: ^#^P=0.001, CIHAng *vs* Norm rats and NormAng rats; 21 days: ^#^P=0.006, CIHAng *vs* Norm rats and ^#^P=0.003, CIHAng *vs* NormAng rats; 28 days: ^#^P=0.0001, CIHAng *vs* Norm rats and NormAng rats (ANOVA followed by LSD for multiple comparisons).

As observed by indirect determination, CIH rats displayed a significant increase in SBP compared with the Norm and NormAng groups from the end of the second week (14 days: CIH *vs* Norm *vs* NormAng: 125.5±7.4 *vs* 100.8±5.3 *vs* 100.0±6.1 mmHg; CIH *vs* Norm: P<0.0001, CIH *vs* CIHAng: P=0.001). By the end of the treatment period with Ang(1-7), SBP was restored, reaching values statistically different from those obtained in the CIH group (28 days: CIH *vs* CIHAng: 145.0±8.2 *vs* 127.0±9.3 mmHg, P<0.0001). No statistical difference in SBP was observed between the Norm and NormAng groups (P>0.05; Figure 1).

### RSNA

**Figure 2:**
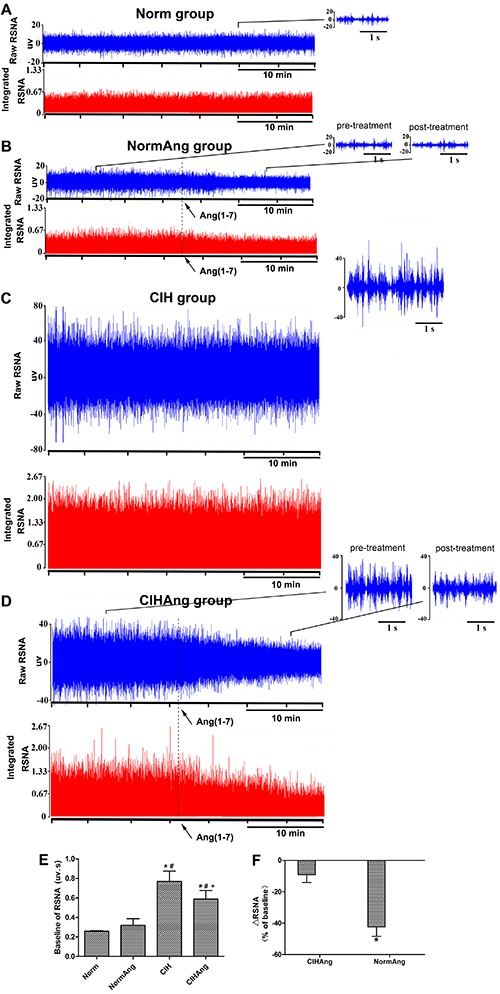
Original recording and integrated renal sympathetic nerve activity (RSNA) in each group. Note the low baseline level of RSNA in normoxia (*A*, Norm and *B*, NormAng), moderate RSNA level in CIHAng(*C*), and enhanced RSNA induced by CIH (*D*). *E*, Baseline levels of each group. *F*, Δ RSNA change compared to baseline after Ang(1-7) administration. Data are reported as means±SE for n=8 rats per group. Norm: normoxia control group. NormAng: Norm and Ang(1-7) supplemented group. CIH: untreated chronic intermittent hypoxia group. CIHAng: CIH and Ang(1-7) supplemented group. *E*, *<0.0001, CIH and CIHAng *vs* Norm; ^#^<0.0001, CIH and CIHAng *vs* NormAng;^+^<0.0001, CIHAng *vs* CIH (ANOVA). *F*, ^*^<0.0001, CIHAng *vs* CIH.

As shown in Figure 2, the RSNA baseline of the CIH group was significantly higher than that of the CIHAng group, which was higher compared with the Norm and NormAng groups. However, there were no significant differences between the Norm and NormAng groups with respect to the baseline. After the acute intervention of Ang(1-7), RSNA was attenuated in the NormAng group. However, this change was significantly greater in the CIHAng group (P<0.05; Figure 2).

### Oxidative stress evaluation in kidney homogenates

**Figure 3:**
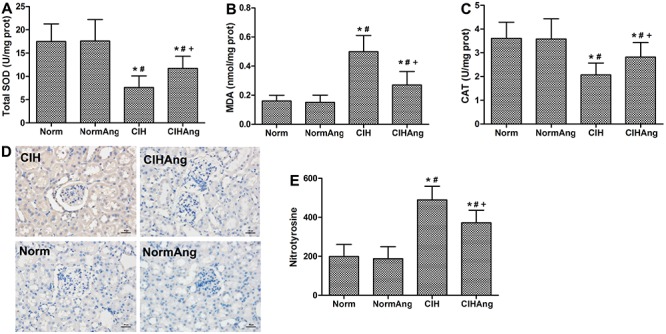
Effects of Ang(1-7) on oxidative stress in renal tissues. *A*, Total superoxide dismutase (SOD) activity; *B*, content of malondialdehyde (MDA), and *C*, level of catalase (CAT). *D*, Immunohistochemistry of nitrotyrosine residues in the kidney of CIHAng rats subjected to chronic treatment with Ang(1-7) (×400). Kidney sections show nitrotyrosine-positive immunostaining in Norm, NormAng, CIHAng, and CIHAng groups. *E*, Quantification of nitrotyrosine in each group. Norm: normoxia control group; NormAng: Norm and Ang(1-7)-supplemented group; CIH: untreated chronic intermittent hypoxia (CIH) group; CIHAng: CIH and Ang(1-7)-supplemented group. Data are reported as means±SE for 8 rats per group. *P<0.05 *vs* Norm; ^#^P<0.05 *vs* NormAng; ^+^P<0.05 *vs* CIH (ANOVA).

As observed in Figure 3A–C, both CAT and SOD renal enzymatic activities were decreased and MDA was increased in the CIH group, indicating a decreased antioxidant capacity in the kidney of this animal model. After chronic treatment with Ang(1-7), a significant decline of MDA and rising CAT and SOD levels were observed. These results confirmed that Ang(1-7) treatment effectively attenuated the state of oxidative stress in the kidneys of CIH rats. Nitrotyrosine content was also determined as an additional parameter of renal oxidative stress in rats. The CIH group displayed increased tissue nitrotyrosine levels, whereas chronic treatment with Ang(1-7) led to a significant reduction of protein nitration levels in the kidney (Figure 3D and E).

### Measurement of renal interstitial fibrosis in CIH

**Figure 4:**
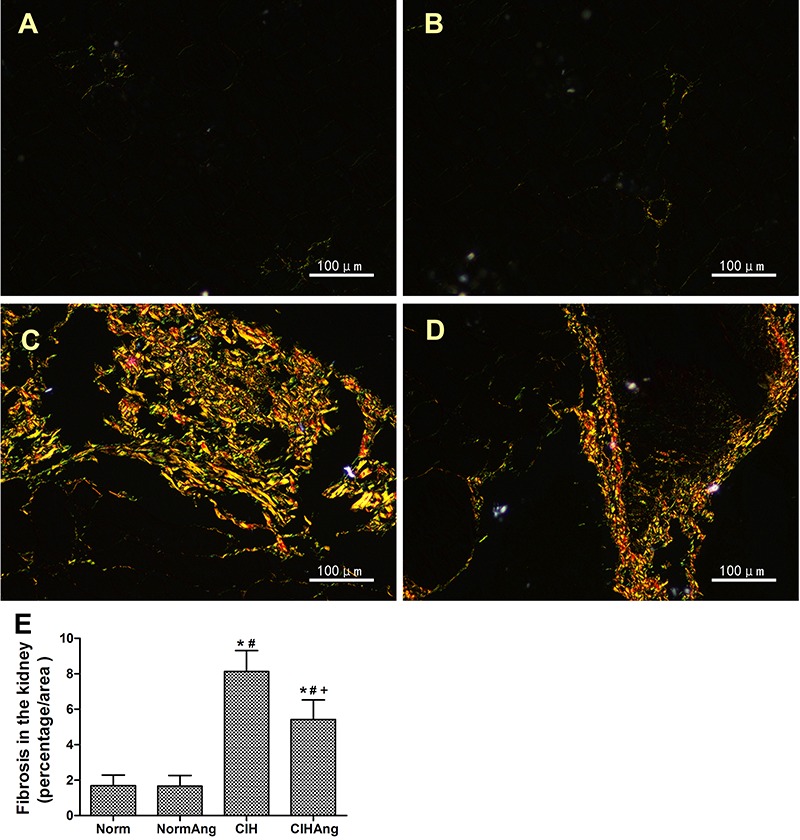
Sirius red staining illuminated with polarized light to illustrate areas of fibrosis in kidneys of chronic intermittent hypoxia (CIH) rats subjected to chronic treatment with Ang(1-7). In Norm (*A*) and NormAng (*B*) groups little collagen expression is observed; in CIH group (*C*), strong collagen expression occurred in renal tissue; in CIHAng (*D*) group, expression intensity was attenuated by Ang(1-7), n=8. Norm: normoxia control group; NormAng: Norm and Ang(1-7)-supplemented group; CIH: untreated CIH group: CIHAng: CIH and Ang(1-7)-supplemented group.

**Figure 5:**
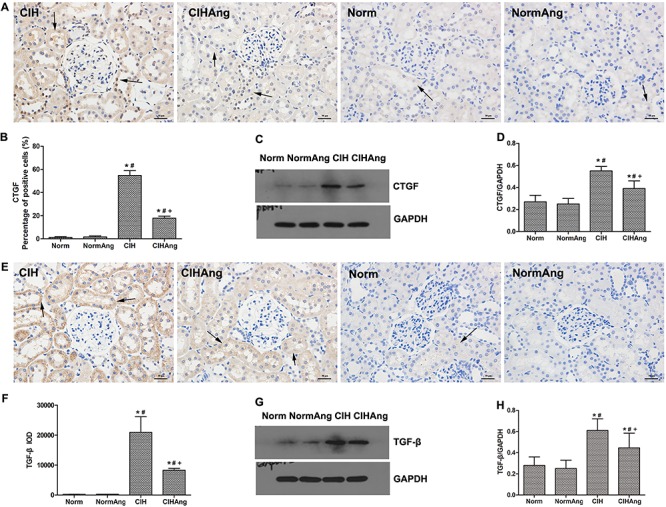
Representative photomicrographs of kidney immunohistochemical analysis (400×) show that in the chronic intermittent hypoxia (CIH) group, CTGF (*A*) was expressed at high levels in glomerular epithelial cells and tubular epithelial cells (dominantly in the nuclei) and TGF-β (*E*) was localized dominantly in the cytoplasm. The supplementation of Ang(1-7) attenuated expression of both the CTGF and TGF-β proteins. Slight expression could be seen in renal tubules and glomeruli, as indicated by the arrows. *B*, Percentage of CTGF positive cells. *C*, Representative images showing the abundance of CTGF and TGF-β (G), assessed by specific immunoblotting analysis in the kidney. Densitometric evaluation of independent western blot of CTGF (*D*) and TGF-β (*H*). *F*, Comparison of integrated optical density (IOD) values among the 4 groups *P<0.006, *vs* Norm; ^#^P<0.006, *vs* NormAng; ^+^P<0.006, *vs* CIH (ANOVA). Data are reported as means±SE. Norm: normoxia control group; NormAng: Normoxia and Ang(1-7)-supplement group; CIH: untreatment CIH group; CIHAng: CIH and Ang(1-7)-supplement group (n=8 per group).

As observed in Figure 4, renal sections subjected to Sirius red staining and illuminated with polarized light indicated that the CIH group presented an increased degree of fibrosis compared with the Norm group. Notably, the CIHAng group chronically treated with Ang(1-7) showed a significant reduction in the expansion of extracellular matrix proteins. This reduction was accompanied by a decreased production of CTGF and TGF-β (Figure 5A, B, E and F: percentage of positive cells for CIH *vs* CIHAng: CTGF=54.7±4.22 *vs* 17.8±1.77%, P<0.0001; IOD: TGF-β=20,906.0±5220.9 *vs* 8275.4±629.2, P<0.0001, respectively). Accordingly, CIH rats displayed an increased abundance of CTGF and TGF-β compared with the Norm group. Ang(1-7) treatment led to a reduction of these markers in the kidney in CIHAng group. Immunostaining results were confirmed by immunoblotting analysis of kidney homogenates (Figure 5C, D, G, and H; CIH *vs* CIHAng: CTGF=0.535±0.08 *vs* 0.378±0.09; TGF-β=0.605±0.07 *vs* 0.445±0.09, respectively). No statistical difference was observed between the Norm and NormAng groups (P>0.05).

### Determination of inflammatory markers in the CIH kidney

**Figure 6:**
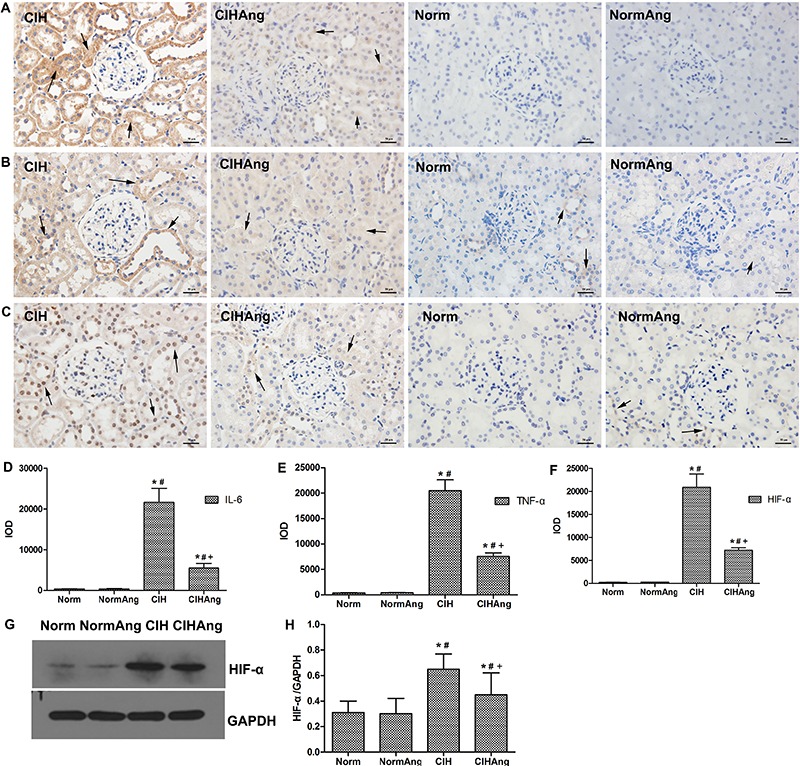
Immunohistochemical expression of IL-6 (*A*), TNF-α (*B*), and HIF-1α (*C*) in rat renal tissue. Representative photomicrographs of kidney immunohistochemical analysis (400×) showing that TNF-α and IL-6 were expressed strongest in renal tubular epithelial cells whereas little expression was observed in glomerular epithelial cells in the chronic intermittent hypoxia (CIH) group. Ang(1-7) supplementation caused a lower expression of TNF-α and IL-6. Slight expression could be seen in renal tubules and glomeruli in the Norm and NormAng groups (arrows). The expression of the HIF-1α protein was high in CIH both in the cytoplasm and nucleus in tubular and glomerular epithelial cells. The administration of Ang(1-7) significantly reduced the expression of HIF-α. Norm and NormAng groups showed little expression of HIF-α in renal tissue. Comparison of integrated optical density (IOD) values for IL-6 (*D*), TNF-a (*E*), and HIF-1α (*F*). Representative images showing the abundance of HIF-α (*G*) assessed by specific immunoblotting analysis in the kidney and its quatification (*H*). Data are reported as the means±SE. *P<0.02, *vs* Norm; ^#^P<0.02 *vs* NormAng; ^+^P=0.001 *vs* CIH (ANOVA). Norm: normoxia control group (n=8); NormAng: Norm and Ang(1-7)-supplemented group (n=8); CIH: untreated CIH group (n=8); CIHAng: CIH and Ang(1-7)-supplemented groups (n=8).

**Figure 7:**
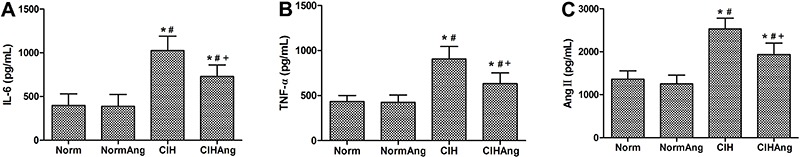
*A*, IL-6, *B*, TNF-α, *C*, Ang II levels in renal tissue. Data are reported as means±SE. Norm: normoxia control group; NormAng: Norm and Ang(1-7)-supplemented group; CIH: untreated CIH group; CIHAng: CIH and Ang(1-7)-supplemented group (n =8 per group). *P<0.01 *vs* Norm; ^#^P<0.01 *vs* NormAng; ^+^P<0.01 *vs* CIH (ANOVA).

The local expression of IL-6, TNF-α, and HIF-α as markers of inflammation was also examined by immunohistochemical, immunoblotting, and ELISA. As observed in Figure 6A–F (IOD: CIH *vs* CIHAng: IL-6=21,607.3±3418.8 *vs* 5532.1±1141.8; TNF-α=20,497.4±2126.2 *vs* 7547.7±696.7; HIF-α=20,883.9±2918.2 *vs* 7199.2±602.1, respectively), the nontreated CIH group presented an increased renal expression of IL-6, TNF-α, and HIF-α compared with the Norm group. The reduction in renal fibrosis was accompanied by a decreased production of local proinflammatory markers. As was observed for other fibrosis markers evaluated, chronic treatment with Ang(1-7) reduced the expression of IL-6 and TNF-α in the CIHAng kidney. This result was confirmed by immunoblotting (Figure 6G and H) and ELISA (CIH *vs* CIHAng: IL-6=1025.1±164.9 *vs* 729.6±130.7 pg/mL, P<0.0001; TNF-α=906.7±137.4 *vs* 632.7 ±119.1, P<0.0001; Figure 7A and B).

### Level of AngII

As shown in Figure 7C, the CIH group displayed a significant increase in AngII levels compared with the Norm and NormAng groups (CIH *vs* Norm *vs* NormAng=2533.66±247.94 *vs* 1362.98±192.45 *vs* 1255.22±197.79 pg/mL, P<0.0001). The AngII level was lower in the CIHAng group (1935.63±266.13 pg/mL) than in the CIH group but higher than in the Norm and NormAng groups (P<0.0001). There were no significant differences between the Norm and NormAng groups (P=0.353).

### Renal cell apoptosis

**Figure 8:**
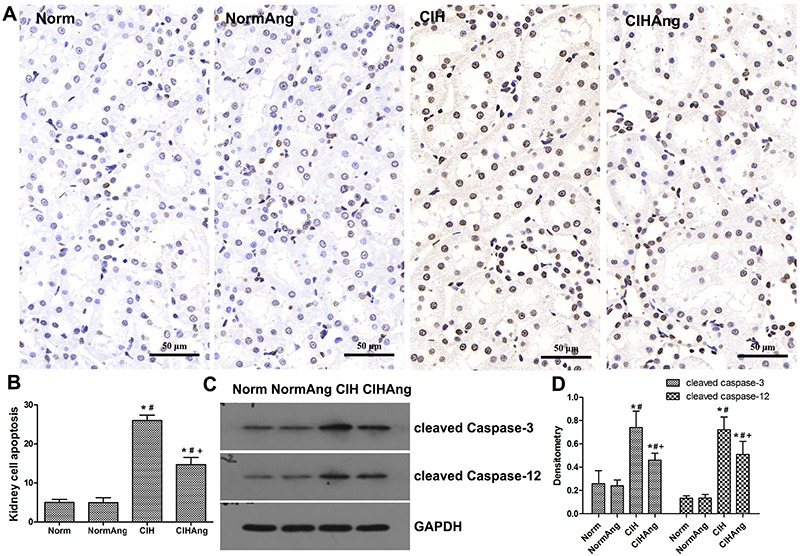
*A*, Representative microscopy images of tubular epithelial cell apoptosis (TUNEL assay, original magnification ×200). *B*, Percentage of TUNEL (+) cells in kidney tissues. *C*, Protein levels of cleaved caspase-3 and cleaved caspase-12 in the four groups. *D*, Densitometric evaluation of the independent western blot. Data are reported as means±SE. Norm: normoxia control group; NormAng: Norm and Ang(1-7)-supplemented group; CIH: untreated CIH group; CIHAng: CIH and Ang(1-7)-supplemented group (n=8 per group). *P≤0.001, *vs* Norm; ^#^P≤0.001, *vs* NormAng; ^+^P≤0.001, *vs* CIH.

TUNEL staining (Figure 8A and B) showed that the percentage of TUNEL-positive cells was higher in the CIH group (25.99±1.38%) than in both Norm (5.01±0.77%) and NormAng groups (4.96±1.27%) (all P<0.05). The ratio of TUNEL-positive cells in the CIHAng group (14.71±1.84%) was lower than in the CIH group but higher than in the Norm and NormAng groups (all P<0.05). There were no significant differences in the ratio of TUNEL-positive cells between the Norm and NormAng groups (P>0.05). The protein levels of cleaved caspase-3 and cleaved caspase-12 (Figure 8C and D) were significantly elevated in the CIH and CIHAng groups compared to the Norm and NormAng groups; however, the levels in the CIH group were higher than those in the CIHAng group (all P<0.05). No differences were observed between the Norm and NormAng groups (P>0.05).

## Discussion

The incidence of OSA is associated with CKD. The prevalence of sleep apnea has been found to be as high as 54-94% in non-dialysis dependent patients with CKD and 30-93% in end-stage renal disease populations. In addition, the prevalence of OSA has been increasing worldwide over recent decades. As OSA is usually associated with a reduction in blood oxygen saturation (1), rats with CIH-induced hypoxia-reoxygenation represent a widely used animal model of human OSA.

Hypoxia and altered O_2_ perfusion are potential players in the development of renal injury. Many of the redox-sensitive cytokines such as IL-6 and TNF-α are proinflammatory and might play a critical role in the initiation and progression of renal injury. In addition, several factors including oxidative stress, fibrosis, and activation of the renin-angiotensin system (RAS) are involved in the pathogenesis of renal injury and lead to the impaired control of RSNA and resistant hypertension. In this study, CIH rats were randomized to receive Ang(1-7) to assess the effect of this treatment on OSA-induced renal injury. We determined that Ang(1-7) induced a protective role and antihyperactive effect in the kidney of CIH animals. First, we demonstrated that CIH could induce hypertension and renal cell apoptosis as well as oxidative stress, inflammation, and fibrosis. Then, we found that Ang(1-7) lowered blood pressure and ameliorated CIH-induced renal injury, reducing the fibrosis and inflammation in the kidney as well as the associated oxidative stress.

Ang(1-7) is formed from Ang II by a prolyl-endopeptidase, prolyl-carboxypeptidase, or ACE2, or directly from Ang I through hydrolysis by a prolyl-endopeptidase and an endopeptidase and is metabolized by ACE to Ang-(1-5). In general, Ang(1-7) opposes the vascular and proliferative effects of Ang II and exerts complex renal actions in chronic renal diseases and hypertension. Xu et al. (19) demonstrated that genetic deletion of the Ang(1-7) receptor Mas produces an extremely complex phenotype that includes increased blood pressure and decreased baroreflex function. Casali et al. (20) reported that the deletion of the Mas receptor is associated with an increase of systolic arterial pressure and with increased blood pressure variability, indicating that the Ang(1-7)/Mas axis is important in the autonomic modulation of arterial pressure. In turn, our results suggest that a chronic subcutaneous perfusion of Ang(1-7) manifested BP-lowering effects under conditions of chronic hypoxia. This concept is further supported by recent findings in other hypertensive models (i.e., in two-kidney, one-clip (2K1C) Goldblatt hypertensive rats and in Ren-2 transgenic rats, a well-defined monogenetic model of hypertension (21,22).

Glomerular or interstitial inflammation is thought to play an important role in the initiation of CKD. Furthermore, the release of inflammatory cytokines has been shown to play a pivotal role in the pathogenesis of OSA-associated renal injury. In the present study, the induction of CIH in rats resulted in a significant increase in IL-6 and TNF-α. Ang II has been shown to have a proinflammatory role in OSA and inhibiting its actions improves inflammation. Consistent with this, Fang et al. (14) demonstrated that deletion of the *Ace2* gene significantly increases cellular inflammation, pro-inflammatory cytokine expression, and apoptosis following ischemia/reperfusion (I/R). Liu et al. (23) have demonstrated that higher ratios of Ang II/Ang-(1-7) increased concentrations of inflammatory factors such as TNF-α at renal tissue. Accordingly, the present results provide strong evidence for an anti-inflammatory effect of Ang(1-7) in the kidney as evinced by its ability to improve the levels of several proinflammatory cytokines (IL-6 and TNF-α) in CIH rats. In addition, we found that the level of Ang II in the CIH group was higher than that in the CIHAng group. Dilauro et al. (24) demonstrated that treatment with Ang(1-7) significantly decreased plasma levels of AngII and hypothesized that Ang(1-7) could promote the increased degradation of Ang II and thereby confer protection in CKD. It was proposed that the activation of the counter-regulatory RAS axis, ACE2-Ang(1-7)-Mas, could oppose the effects of the ACE-Ang II-AT1 axis and prevent or reverse organ damage.

Intermittent hypoxia (IH) is believed to induce oxidative stress, which contributes to renal injury. Welch et al. (25) reported that the administration of Ang II to rats using osmotic minipumps resulted in hypertension and elevated oxidative stress. In addition, reactive oxygen species (ROS), whether mitochondrial- or cell membrane-derived, might also be responsible for the activation of classical RAS components, contributing further to the compromise of renal functions. Sun et al. (26) demonstrated that when mice were exposed to long-term IH, the kidney became incapable of tolerating IH-induced changes, reflected by a second increase of renal inflammation along with lipid peroxidation, cell death, and fibrosis. In comparison, our study found that the level of oxidative stress increased in the kidney tissue in the CIH group. This finding is also in accordance with other reports that CIH caused the production of ROS (27–29). Wysocki et al. (30) demonstrated that the genetic ablation of ACE2 in mice was associated with increased kidney ROS owing to elevated levels of NADPH oxidase activity. Other studies demonstrated that both apoptosis and oxidative stress, two processes that are associated with I/R and are influenced by Ang II, were exacerbated by the loss of the *Ace2* gene (31,32) and that repetitive episodes of hypoxia/reoxygenation that are associated with the transient cessation of breathing during sleep in OSA resemble I/R injury (33). Consistent with these findings, the present study demonstrated that the enhanced oxidative stress in CIH appeared to be significantly inhibited by Ang(1-7) treatment. Thus, it can be hypothesized that the ability of Ang(1-7) to notably inhibit oxidative stress renders a protective effect on CIH rats.

A hypoxic environment accelerates fibrosis, which results in a loss of peritubular capillaries that correlates with the deterioration in renal function. In addition, tubulointerstitial hypoxia stimulates the production of collagen, an indicator of increased fibrogenesis. In 1998, Fine et al. (34) suggested chronic hypoxia served as the common mediator of the development of progressive renal disease, with hypoxia acting as the transmitter of glomerular injury to the tubulointerstitium. The stipulated “chronic hypoxia hypothesis” suggested that the hypoxic milieu triggers a fibrotic response and the development of fibrosis in tubulointerstitial cells. In this model, hypoxia causes a decrease in anti-fibrotic factors in parallel with an increased expression of fibrogenic factors, e.g., TGF-β, CTGF and Ang II (35,36). According to Kagami et al. (37), Ang II and ROS play an important role in cellular signaling and the activation of genes of extracellular matrix proteins, such as collagens and fibronectin via TGF-β (37). By affecting the tissue adjacent to capillaries and nephrons, the fibrotic response, in turn, exacerbates the hypoxia and fibrosis, leading to a vicious cycle (38). A protective role for Ang(1-7) in this process was evidenced in Mas receptor knockout mice, which display renal dysfunction associated with increased interstitial fibrosis and the upregulation of mRNA for TGF-β (39,40). In accordance with these reports, in the present study we determined that the chronic subcutaneous perfusion of Ang(1-7) ameliorated renal fibrosis and improved the hypoxic condition in the kidney of CIH animals.

In summary, our findings indicated that Ang(1-7) lowered blood pressure and induced a protective role in the kidneys of CIH animals and that these effects appeared to be mediated, at least in part, by inhibiting the release of proinflammatory cytokines and ameliorating oxidative stress and fibrosis. These results highlight the therapeutic potential of enhancing Ang(1-7) action as a novel therapy for CIH-induced target organ damage.
